# Serpin peptidase inhibitor (*SERPINB5*) haplotypes are associated with susceptibility to hepatocellular carcinoma

**DOI:** 10.1038/srep26605

**Published:** 2016-05-25

**Authors:** Shun-Fa Yang, Chao-Bin Yeh, Ying-Erh Chou, Hsiang-Lin Lee, Yu-Fan Liu

**Affiliations:** 1 Institute of Medicine, Chung Shan Medical University, Taichung, Taiwan; 2 Department of Medical Research, Chung Shan Medical University Hospital, Taichung, Taiwan; 3 Department of Emergency Medicine, School of Medicine, Chung Shan Medical University, Taichung, Taiwan; 4 Department of Emergency Medicine, Chung Shan Medical University Hospital, Taichung, Taiwan; 5 School of Medicine, Chung Shan Medical University, Taichung, Taiwan; 6 Department of Surgery, Chung Shan Medical University Hospital, Taichung, Taiwan; 7 Department of Biomedical Sciences, College of Medical Science and Technology, Chung Shan Medical University, Taichung, Taiwan; 8 Division of Allergy, Department of Pediatrics, Chung Shan Medical University Hospital, Taichung, Taiwan

## Abstract

Hepatocellular carcinoma (HCC) represents the second leading cause of cancer-related death worldwide. The serpin peptidase inhibitor *SERPINB5* is a tumour-suppressor gene that promotes the development of various cancers in humans. However, whether *SERPINB5* gene variants play a role in HCC susceptibility remains unknown. In this study, we genotyped 6 SNPs of the *SERPINB5* gene in an independent cohort from a replicate population comprising 302 cases and 590 controls. Additionally, patients who had at least one rs2289520 C allele in *SERPINB5* tended to exhibit better liver function than patients with genotype GG (Child-Pugh grade A vs. B or C; *P* = 0.047). Next, haplotype blocks were reconstructed according to the linkage disequilibrium structure of the *SERPINB5* gene. A haplotype “C-C-C” (rs17071138 + rs3744941 + rs8089204) in *SERPINB5*-correlated promoter showed a significant association with an increased HCC risk (AOR = 1.450; *P* = 0.031). Haplotypes “T-C-A” and “C-C-C” (rs2289519 + rs2289520 + rs1455555) located in the *SERPINB5* coding region had a decreased (AOR = 0.744; *P* = 0.031) and increased (AOR = 1.981; *P* = 0.001) HCC risk, respectively. Finally, an additional integrated *in silico* analysis confirmed that these SNPs affected *SERPINB5* expression and protein stability, which significantly correlated with tumour expression and subsequently with tumour development and aggressiveness. Taken together, our findings regarding these biomarkers provide a prediction model for risk assessment.

Hepatocellular carcinoma (HCC) is one of the most common malignancies worldwide and represents the second and sixth leading cause of cancer related death among males and females in 2012, respectively^1^. According to GLOBOCAN 2012, 859,400 new cases of primary liver cancer occur each year, with 70% to 90% being HCC^1^. HCC carcinogenesis is a multistep and complex process^2^. Well-documented risk factors include major etiologic agents such as chronic hepatitis B virus (HBV) or hepatitis C virus (HCV) infection^3^ and aflatoxin B1^2^. Recent studies have shown that genetic mutations and environmental factors may increase the severity of the hepatic inflammation that contributes to cell differentiation, proliferation and the deposition of connective tissue, which are necessary for the development of HCC^4^,^5^,^6^.

The HCC prediction score reported by Wang *et al.* is a simple scoring system that uses clinical and laboratory parameters such as age, serum albumin level, serum bilirubin level, HBV and the presence of liver cirrhosis^7^. Elderly patients are at a higher risk for developing HCC even after stratification by stage of hepatic fibrosis^8^. The age-specific incidence rates also reflect the differences in the natural history of HBV- and HCV-related cases of liver cancer^2^. For example, in Taiwan the mean age of the onset of HBV-positive liver cancer is 52.5 years, which is 10 years younger than the average age at the onset of HBV-negative cases^9^. Aging is becoming one of the major risk factor for HCC and thus represents a real problem for the future, particularly in Asian countries^10^.

*SERPINB5*^11^ [also known as maspin (mammary serine proteinase inhibitor)] belongs to the serpin superfamily of proteins and has been grouped with the *ov-serpin* subfamily (clade B)^12^. *SERPINB5* is a tumour suppressor that binds directly to extracellular matrix components, suggesting that the surface binding interaction is responsible for the inhibition of tumour-induced angiogenesis, invasion and metastatic spread^13^. The *SERPINB5* mRNA and protein are produced in normal mammary epithelial cells. Gene expression is partially down-regulated in primary cell lines, and the loss of *SERPINB5* expression is correlated with increasing malignancy in several tumours, such as breast^14^, prostate^15^, thyroid^16^ and skin^17^. *SERPINB5* is an important senescence-associated marker and prognostic tumour suppressive factor in the complex carcinogenic process^18^,^19^.

Based on several known serpin structures including *SERPINB5*, serpins have the capacity to bind co-factors and undergo serine protease-induced conformational changes in which the serpin reactive centre loop (RCL) develops a non-standard hinge region that is able to trap and inhibit serine proteases^20^. Eventually, *SERPINB5* adopts the native serpin fold consisting of nine α-helices and three β-sheets. Strand 4A is in close vicinity to the RCL and may play an important role in a covalent bond interaction with the catalytic site of the target protease^21^.

Although the development of HCC may take 20 to 50 years, early detection of this cancer is seldom available due to the lack of reliable markers. Therefore, the disease runs a largely asymptomatic disease course until it is too advanced for successful treatment^22^. Aberrations in some genes may be responsible for certain clinical features of HCC^23^. For instance, differences in the *SERPINB5* expression level have been demonstrated between precancerous and malignant lesions^18^. However, the associations between *SERPINB5* variants and HCC risk and prognosis have been poorly investigated. Gene expression is affected by SNPs located within the promoter or other regulatory regions of the gene. Thus, six *SERPINB5* SNPs located in two linkage disequilibrium (LD) blocks were genotyped to perform a haplotype-based association analysis in a case control study of the Taiwanese population to evaluate HCC susceptibility.

## Results

### Study Population

A total of 892 participants, including 302 HCC cases and 590 controls were successfully genotyped for further analysis. The demographic characteristics including mean age, gender, alcohol consumption, tobacco consumption and disease stage of are shown in Table 1. No significant differences existed between groups of alcohol consumption (*P* = 0.809) and tobacco use (*P* = 0.395) in the healthy controls and patients with HCC. Whereas, the mean age (Control: 51.11 ± 14.97 years; HCC: 63.01 ± 11.78 years; *P* < 0.001) were significantly lower comparing the matched-free cohort from the general population, female sex (Control: 18.1%; HCC: 29.5%; *P* < 0.001) was more prevalent (Table 1). Overall, there was an increasing risk of HCC observed with increasing age.

### Frequency Distribution of SERPINB5 Alleles and their Associations with HCC

Six SNPs in the *SERPINB5* gene were genotyped in HCC patients and the healthy controls using Hardy-Weinberg equilibrium (*P *> 0.05). Table 2 summarized the basic characteristics of *SERPINB5* SNPs in the study population which indicated the highest distribution frequency for rs17071138, rs3744941, rs8089104, rs2289519, rs2289520 and rs1455555 loci of *SERPINB5* gene were T/C, C/T, C/T, T/C, G/C and A/G, respectively, in both HCC patients and healthy control subjects. According to the adjusted odds ratios (AORs) with their 95% confidence interval (CI) with multiple logistic regression model for HCC of *SERPINB5* gene polymorphism, only rs2289520 CC or GC + CC presented a significant (*P* < 0.05) lower risk of 0.247-fold (95% CI, 0.113–0.543) and 0.666-fold (95% CI, 0.477–0.929) have HCC compared with their corresponding wild-type homozygotes after adjusting confounding factors (Table 2). To explore the impact of polymorphic genotype rs2289520 of *SERPINB5* on clinic-pathological development of HCC, we further classified the HCC patients and heathy control subjects into two subgroups each: one subgroup with at least on polymorphic alleles (GC + CC) and the other subgroup with homozygous wild type alleles (GG). Patients with at least one polymorphic C allele of *SERPINB5* rs2289520 was found to be significantly associated with high Child-Pugh grade B/C [odds ratio (OR) = 0.527, 95% CI, 0.328–0.996, *P* = 0.047] that significant predictors of poor survival^24^ (Table 3). In addition, we also further analyzed the potential between six independent SNP locus of *SERPINB5* and the levels of several serum markers such as AFP, aspartate transaminase (AST) and alanine transaminase (ALT) values and AST/ALT ratio. As a consequence, no significantly difference in the serum levels of these markers was detected between patients who possess at least on polymorphic allele and those who do not for any of the *SERPINB5* SNPs examined (Table S2).

### Haplotype analysis of the SERPINB5 gene

All of the subjects were genotyped for a total of 6 tag SNPs that were selected to cover (*r*^*2 *^≥ 0.70) most of the SNPs located in a 32 kb region, including the *SERPINB5* gene (28 kb), its promoter (2 kbp) and the 3′-untranslated region (3′-UTR, 2 kbp). *SERPINB5* polymorphisms were further characterized using LD and haplotype analyses. LD was determined pairwise among all 45 SNPs and the haplotype structure of *SERPINB5* gene was analyzed (D’ and *r*^*2*^) according to the 1000 Genomes Project data from the East Asian population (CHB + JPT, Fig. S1)^25^. Haplotype blocks divided by D’ confidence interval method, D’ value of 95% CI 0.70~0.98 in adjacent SNPs were classified as the same haplotype block. Two LD blocks were detected by Solid Spine^26^ of haplotype phasing techniques. Block1 (15 kb) consisted of 3 closely selective SNPs showed strong linkage, rs17071138, rs374491 and rs8089104, in promoter of *SERPINB5* (Fig. S1). Block2 (17 kb) included two completely linked selective SNPs, rs2289519 and 2289520. Additional, weak linkage between rs2289520 and rs14555556 was observed in coding region (Table S1).

**Figure 1:**
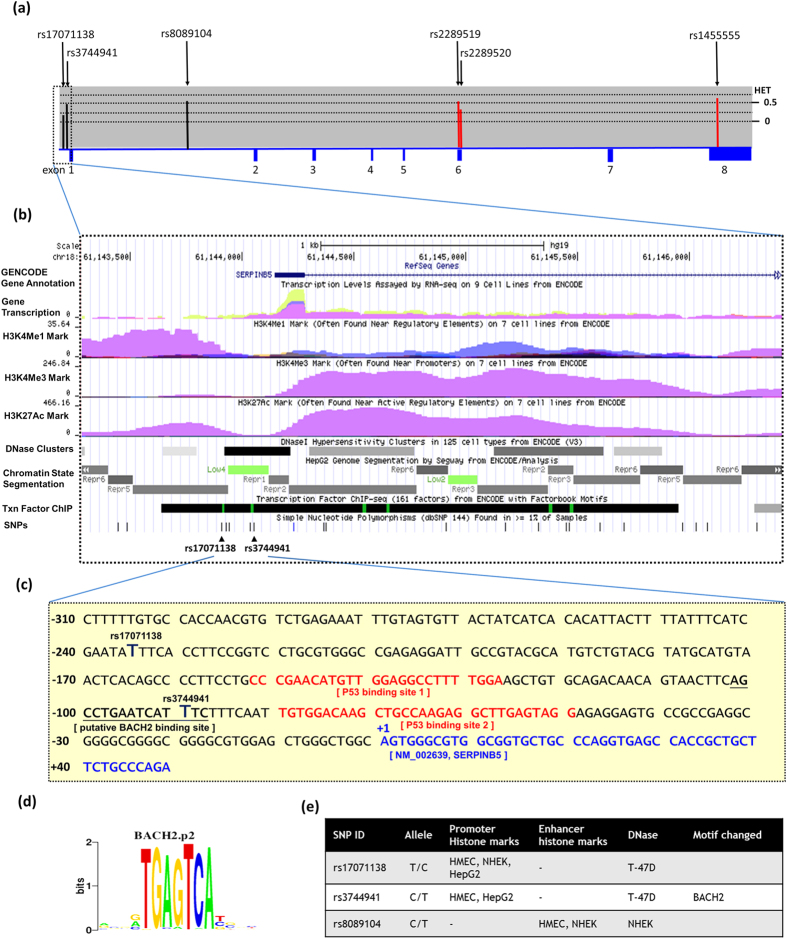
Exon, intron, gene features and SNPs analysed of the human *SERPINB5* (NM_002639.4). (**a**) Exons are indicated by the filled blue boxes and are numbered from 1 to 8 from chromosome positions chr18:61,144,144 to 61,172,318 (reference genome GRCh37.p13). The lower panel shows population-specific heterozygosity frequencies of these polymorphisms in the East Asian population. SNPs are indicated by black arrows and labelled with the SNP ID number. (**b**) Expanded view of ENCODE data for the promoter block containing rs3744941 and rs17071138 using UCSC genome browser. The H3K4Me1, H3K4Me3, and H3K27Ac tracks show the genome-wide levels of enrichment of the mono-methylation of lysine-4, tri-methylation of lysine-4, and acetylation of lysine-27 of H3 histone protein, respectively, as determined by the ChIP-seq assays. Chromatin State Segmentation track displays chromatin state segmentation by integrating ChIP-seq data using a HMM for HepG2 HCCs. Chromatin state regions predicted for the promoters and transcribed are highlighted with red and green, respectively. DNase cluster tracks show DNase hypersensitivity areas. Tnx Factor tracks show transcription factor binding to DNA based on ChIP-seq and CpG islands. **(c)** +1 represents the transcription initiation site of the *SERPINB5* and is highlighted in blue font. Consensus residues of the core P53 (RRRCWWGYYY N[0–13] RRRCWWGYYY) are indicated with red fonts, where R denotes A or G, W denotes A or T, and Y denotes C or T. The putative BACH2 binding site indicated with an underline and the position of selective SNPs revealed with bold font. **(d)** Motif logo of the BACH2.p2 consensus sequence from the SIB EPD. **(e)** Regulatory annotations of SNPs within the LD blocks containing *SERPINB5* rs3744941. Multiple regulatory motifs were predicted to be affected.

Finally, a haplotype-based association study was performed to show the association between *SERPINB5* haplotype and risk of HCC (Table 4). The block1 of promoter SNPs constituted virtually three haplotypes of approximately equal frequencies in control subjects (42.8%, 35.4% and 11.6%), but one haplotype in the HCC cases, “C-C-C”, was associated with increased susceptibility to HCC (OR = 1.450; 95% CI, 1.039–2.025, *P* = 0.029). The expression of the *SERPINB5* has been demonstrated to be under the control of the oncogenic transcription factors TP53^27^ and TP63^28^. Moreover, the 300 bp promoter sequence containing the HCC-risk-associated haplotype was identified the putative functional role of rs3744941 by the functional annotations in the Encyclopedia of DNA elements (ENCODE) data^29^ (Fig. 1). We determined the rs3744941 was situated at a locus with Transcription factor (TF) binding, histone modification patterns, DNA hyposensitivity, and CpG islands that were characterized as promoter or enhancers in several cell type (Fig. 1b). The effect of rs3744941 may be attributed to the suboptimal BACH2 binding site^30^ (Fig. 1b) surrounding the upstream of the predicted transcriptional start site of human *SERPINB5* gene (Fig. 1b), which enable the modulation of initiation rates in response to the transcriptional status. In addition, the Genome-Tissue Expression (GTEx) database showed a statistically significant down regulation of *SERPINB5* mRNA expression in whole blood of rs17071138 variant genotype (TC) compared with wild-type homozygous genotype (TT, *P* = 0.046, Fig. S2a). A similar result was also found for rs3744941 in the muscle and skeletal tissue (Fig. S2b). Accordingly, these promoter SNPs reduced BATH2 binding site or at least decreased TF binding affinity in the HCC-risk haplotype with reduction gene expression, and increase the susceptibility to HCC (Table 4).

### Protein structural modeling of human SERPINB5

**Figure 2:**
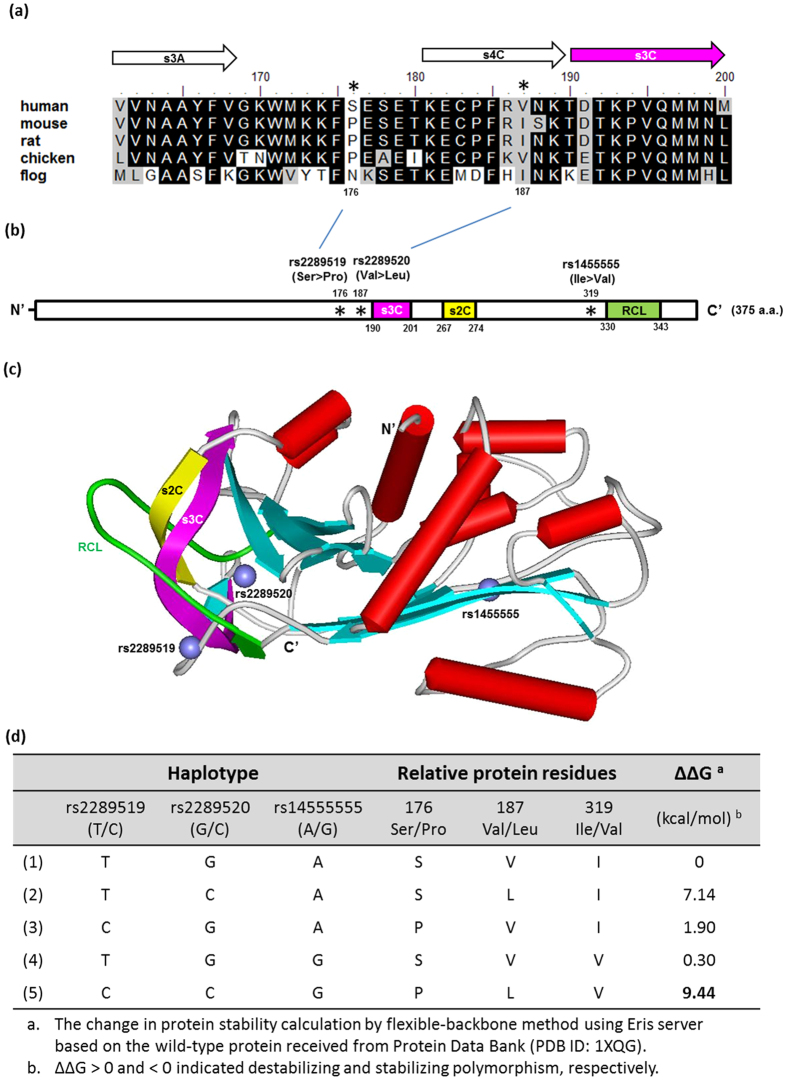
Structural characterization of human *SERPINB5*. (**a**) The selected variant regions between rs2289519 and rs2289520 of five maspin-like proteins including human (NP_002630.2), mouse (NP_033283.1), rat (NP_476449.1), chicken (XP_418986.3) and frog (NP_001011282.1) using a truncated multiple sequence format. The secondary structure of *SERPINB5* is shown above the alignment, and the numbering is based on the human *SERPINB5*. Arrows represent β-strands and cylinders indicates α-helices. **(b)** Schematic representation of the overall human *SERPINB5* protein. Domain symbols are drawn approximately to scale. The rectangles represent the key secondary structures [s2C (yellow) and s3C (pink)] that provided stabilizing interactions with the RCL (green). The positions of rs2289519, rs2289520 and rs1455555 are indicated by asterisks. **(c)** Ribbon diagram (ViewerLite 5.0) showing he 3D structure of human *SERPINB5* (PDB accession number: 1XQG). The N-terminal and C-terminal ends are indicated with N’ and C’, respectively. The purple sphere indicated the Cα carbon of the germline variants characterized in this study. **(d)** Protein stability estimation of *SERPINB5* SNP haplotype variation using a structure-based approach. The free energy of the protein is a weighted sum of the van der Waals, solvation, hydrogen bonding, and backbone-dependent statistical energies.

Haplotype block2 spanning the region of 17 kb coding region of *SERPINB5* containing the HCC-risk-associated nonsynonymous SNPs (nsSNP) have been found in at least 60 residues (http://www.ncbi.nlm.nih.gov/SNP/ snp_ref.cgi?locusId = 5268), including rs2289519, rs2289520 and rs1455555 (Table S1), although single SNP has not significantly increase susceptibility associated with HCC. But, haplotype analysis showed that the “C-C-G” haplotype (corresponding to haplotype block2) increase the risk of HCC (OR = 1.981; 95% CI, 1.313–2.989, *P* = 0.001), while the “T-C-A” haplotype decreased the risk (OR = 0.744; 95% CI, 0.569–0.974, *P* = 0.031) in this case-control study (Table 4). Considering the essential role of *SERPINB5* in various model disease, we performed *in silico* profiling of evolution-based and homology three-dimensional molecular model of the human *SERPINB5* RCL domain (amino acid residues 330–343). Multiple alignment of the deduced amino acids sequence revealed that inter-species amino acid conservation from humans thought flog at the 40-residues stretch of *SERPINB5*. Figure 2a, positions 179 and 187 of *SERPINB5* (the positions of the amino acid substitution corresponding to rs2289519 and rs2289520, respectively). As see several amino acids appeared in these positions caused a conserved change from a polar acid to a slightly polar amino acid, with increased hydrophobicity. These variants are positioned within the functional RCL of the *SERPINB5* gene (Fig. 2c).

In order to understand how the polymorphism could affect protein structure, we further analyzed the effect of structural adjacent variants bearing three nsSNPs (S179P, V191L and I322V) from the haplotype block2 were developed based on published crystal structures of normal *SERPINB5*^31^ (protein data bank, PDB ID: 1XQG, Fig. 2c). Based on physico-chemical properties, change in free energy score (∆∆G), we evaluate functional consequences of deleterious nsSNPs in RCL domain on the protein stability using Eris server^32^. The nsSNPs rs2289519 (S176P) and rs22289520 (V197L) maps on to loop of s3C β sheet and s4C β sheet of *SERPINB5* relatively near the RCL domain is thought to mediate protein activity, respectively (Fig. 2c). Further, from our study, it is clear that the polymorphic amino acids different in size compared to wild-type protein. The notable change of the wild-type buried amino acids were altered and may result in empty space in protein, suggesting that the risk-associated haplotype (C-C-G) correlate to rs2289519-rs2289520- rs1455555 probably alter the catalytic activity of *SERPINB5* protein (Fig. 2d).

## Discussion

Several studies have suggested that chromosome 18q21 contains a tumor suppressor gene involved in multiple tumor types^33^,^34^,^35^. Recent studies have reported that *SERPINB5* polymorphism is associated with the susceptibility to several carcinomas including gastric^36^, lung^37^, bladder^38^, colorectal^39^, and breast^14^ cancers. We hypothesize that genetic variants of the *SERPINB5* may influence clinical outcomes in localized HCC patients. Six SNPs were included in the present case-control study design. One of the SNPs (rs2289520) is located in exon 1 of *SERPINB5*. Our data reveal an increased risk of HCC among patients with the *SERPINB5* polymorphic rs2289520 C/C compared with those with homozygous G/G. Only few studies have examined the functional role of rs2289520^40^, and we present additional evidence for a role of *SERPINB5* in HCC, as elevated *SERPINB5* gene was associated with more aggressive cancers and poorer clinical outcomes.

Further, two SNP haplotypes located on the *SERPINB5* promoter and protein coding regions have been clinically examined probably because a putative BASH2 binding site and RCL domain, typically influences the alternative translation expression efficiency and protein stability, respectively. Although directly testing this hypothesis was beyond the scope of the current study, evidence suggests that decreased promoter activity and protein stability associated susceptibility to HCC, suggesting that *SERPINB5* downregulation is associated with increase susceptibility to HCC. Jang *et al.* showed the polymorphic variant (rs2289518) associated with the cell apoptosis, colony formation and *in vivo* tumor formation^41^. Hence, the present analyses increased our understanding of naturally occurring *SERPINB5* variants, a lesion category that, although not infrequent, has been relatively neglected in terms of exploring the underlying pathogenic mechanisms. An improved understanding of these variants is a prerequisite for developing therapeutic approaches that can eventually ameliorate the clinical phenotype in patients harboring the corresponding lesions.

Surveillance should be offered to patients with a high risk of developing HCC. Although biomarkers are not widely accepted as important clinical tools, they contribute valuable information for the management of patients with HCC, with regards to surveillance, diagnosis, evaluation of treatment efficacy, and prediction of outcomes Comparing the levels of clinical pathological markers, such as AFP, AST, and ALT, which partially reflects the body function and nutrition status, no significant difference between the wild-type and polymorphic genotypes of each *SERPINB5* SNPs in HCC patients was observed (Table S2). HCC is usually diagnosed in cirrhotic patients (60–80%). Nevertheless, significant (*P* < 0.05) pathological characteristics of high Child-Pugh grade B/C appeared, a significant predictor of poor survival outcome in patients with HCC^42^, in the cirrhotic HCC patients with at least one polymorphic C allele of *SERPINB5* rs2289520 genotypes (Table 3). These polymorphic markers may further improve the prediction to evaluate biological status and background liver function.

In conclusion, this study comprises a comprehensive effort in medical information and conducting additional bioinformatics analyses of a high number of patients provided comprehensive evidence of *SERPINB5* polymorphism in HCC. Our results suggest that the *SERPINB5* polymorphic promoter SNPs and nsSNPs in the *SERPINB5* is associated with clinical statuses and susceptibility to HCC. The co-effects of *SERPINB5* polymorphism in translational and protein level markedly facilitate HCC development. Overall, our analyses provide deeper insights into naturally occurring haplotype-based variants. Characterizing the molecular basis of mutations in cancer cells provides insight into tumorigenesis and accurate biomarkers on such types of variant are required for developing optimal therapeutic approaches that can eventually ameliorate the clinical phenotype in patients harboring the corresponding lesions.

## Materials and Methods

### Description of the Enrolled Participants

This hospital-based case control study recruited 302 (213 men and 89 women; mean age = 63.01 ± 11.78 years) HCC patients between 2007 and 2012 at the Chung Shan Medical University Hospital, Taiwan. The HCC diagnosis was based on the criteria specified in the national guidelines for HCC. Specifically, liver tumours were diagnosed by histology or cytology irrespective of the α-fetoprotein (AFP) titre after computed tomography or magnetic resonance imaging data showed at least one of the following: (1) at least one liver mass ≥2 cm in diameter; (2) early enhancement and AFP levels ≥400 ng/ml; or (3) early arterial phase-contrast enhancement plus early venous phase-contrast washout regardless of the AFP level. During the same study period, the 590 ethnic group-matched individuals (483 men and 107 women; mean age = 50.11 ± 14.97 years) were enrolled as these subjects received a physical examination at the same hospital. These control groups had neither self-reported history of cancer of any sites. Personal information and characteristics collected from the study subjects using interviewer-administered questionnaires contained questions involving demographic characteristics and the status of cigarette smoking and alcohol drinking. HCC patients were clinically staged at the time of diagnosis according to the tumour, node and metastasis (TNM) staging system of the American Joint Committee on Cancer^43^. Liver cirrhosis was diagnosed by liver biopsy, abdominal sonography, or biochemical evidence of liver parenchymal damage with endoscopic oesophageal or gastric varices. The patients’ clinicopathological characteristics, including clinical staging, tumour size, lymph node metastasis, distant metastasis, reactivity with an antibody against HCV (anti-HCV), liver cirrhosis, AFP, AST and ALT levels, were verified by chart review. Whole blood specimens collected from the controls and HCC patients were placed in tubes containing ethylenediaminetetraacetic acid (EDTA) and then immediately centrifuged and stored at −80 °C. Before commencing the study, approval was obtained from the Institutional Review Board of Chung Shan Medical University Hospital, and informed written consent was obtained from each individual (CSMUH No:CS15099). All participants provided written consent, and the Chung-Shan Medical University Hospital ethics committees approved the research protocol and informed consent was obtained from all subjects. All the methods applied in the study were carried out in accordance with the approved guidelines.

### SNP Selection and Genotyping

Genomic DNA was isolated from the peripheral blood using the QIAamp DNA blood mini kit (Qiagen, Valencia, CA, USA). The final preparation was stored at −20 °C, quantified by measurement of the optical density at 260 nm and used as the polymerase chain reaction (PCR) template. Genotyping of 6 *SERPINB5* SNPs (rs17071138, rs3744941, rs8089104, rs2289519, rs2289520, and rs1455555; Fig. 1) with minor allele frequencies >5% in the HapMap Chinese Han Beijing (CHB) population was performed by the TaqMan SNP genotyping assay (Applied Biosystems, Foster City, CA, USA)^44^. A total of six SNPs included promoter region SNPs, rs17071138 and rs3744941; intron region, rs8089104, non-synonymous SNPs, rs2289519, rs2289520, and rs1455555 of the gene. SERPINB5 rs17071138, (assay IDs: C_33627662_20), rs3744941 (assay IDs: C_27493638_10), rs8089104 (assay IDs: C_29202434_30), rs2289519 (assay IDs: C_22274204_10), rs2289520 (assay IDs: C_22274205_10), and rs1455555 (assay IDs: C_8932279_10) polymorphisms were assessed using an ABI StepOnePlus TM Real-Time PCR System and analyzed using SDS v3.0 software (Applied Biosystems, Foster City, CA). A genotyping fluorescence-based TaqMan SNP assay was demonstrated to be suitable for the analysis^45^. The final volume for each reaction was 10 μL and contained 5 μL of the TaqMan Universal PCR Master Mix, 0.25 μL of the primer/TaqMan probe mix, and 10 ng of genomic DNA. The real-time PCR reaction consisted of an initial denaturation step at 95 °C for 10 minutes followed by 40 cycles consisting of 92 °C for 15 sec. and 60 °C for 1 min. The fluorescence level was measured with the Applied Biosystems StepOne Real-Time PCR System (Applied Biosystems). Allele frequencies were determined by the ABI SDS software. Genotyping was repeated on a random 10% of the sample to confirm the results of the original run. For each assay, appropriate controls (non-template and known genotype) were included in each typing run to monitor reagent contamination and as a quality control. To validate the real-time PCR results, approximately 5% of the assays were repeated and several cases of each genotype were confirmed by DNA sequencing analysis.

### Bioinformatics Analysis

Several semi-automated bioinformatics tools to assess whether SNPs or their linked genetic variants were associated with a putative function that might affect patient outcomes. HaploReg^46^ v4 and the Genotype-Tissue Expression (GTEx) database^47^ from the Encyclopedia of DNA Elements (ENCODE) project^48^ were used to identify the regulatory potential on candidate functional variants to examine the particular tracks of interest, such as TF-ChIP signals, DNase peaks, DNase footprints and predicted DNA sequence motifs for TFs. The GTEx data were used to identify the correlations between SNPs and whole-blood-specific gene expression levels. The publically available cBioPortal for Cancer Genomics^49^ and UCSC Cancer Genomics Browser^50^ for hepatocellular adenocarcinomas were utilized to analyse *SERPINB5* gene expression, DNA methylation, molecular features, and clinical outcomes

### Sequence alignment and Protein structure

A multiple sequence alignment was generated using the CLUSTALX package with a standard point accepted mutation series protein weight matrix. Five *SERPINB5* orthologous protein sequences were obtained from the NCBI gene database, and the key residues were identified based on the alignment as previously described for the RCL domain and secondary structure, respectively^21^. The 3D structural model of human *SERPINB5* (PDB ID 1XQG) was downloaded from the RCSB PDB database^51^.

### Statistical analysis

The Hardy-Weinberg equilibrium was assessed using a Chi-square goodness-of-fit test for bi-allelic markers. The Mann-Whitney U test and Fisher’s exact test were used to compare differences in the distribution of age and demographic characteristics between the controls and HCC patients. ORs with 95% confidence intervals (CIs) were estimated using logistic regression models. AORs with 95% CIs were used to assess the association between genotype frequencies with HCC risk and clinical factors. *P* values less than 0.05 were considered significant. The data were analysed with the SPSS 12.0 statistical software (SPSS Inc., Chicago, IL, USA). Linkage disequilibrium coefficients [D’ = D/D_max_ (or D/D_min_ if the D’ value was negative)] were assessed for pairs of alleles between the two sites of *SERPINB5* polymorphisms, and haplotype blocks were defined using the default setting of the Haploview software^26^. We estimated the common haplotypes with PHASE version 2.1. A likelihood ratio test was used to perform a global test of association between all haplotypes and HCC occurrence.

## Additional Information

**How to cite this article**: Yang, S.-F. *et al.* Serpin peptidase inhibitor (*SERPINB5*) haplotypes are associated with susceptibility to hepatocellular carcinoma. *Sci. Rep.* **6**, 26605; doi: 10.1038/srep26605 (2016).

## Supplementary Material

Supplementary Information

## Figures and Tables

**Table 1:** The distributions of demographical characteristics and clinical parameters in 590 controls and 302 patients with HCC.

Variables	Controls (N = 590)	Patients (N = 302)	*P*value^†^
Age (yrs)	Mean ± S.D.	Mean ± S.D.	
	50.11 ± 14.97	63.01 ± 11.78	*P* < 0.001
Gender	n (%)	n (%)	
Male	483 (81.9%)	213 (70.5%)	*P* < 0.001
Female	107 (18.1%)	89 (29.5%)	
Alcohol consumption			
No	378 (64.1%)	191 (63.2%)	*P* = 0.809
Yes	212 (35.9%)	111 (36.8%)	
Tobacco consumption			
No	367 (62.2%)	179 (59.3%)	*P* = 0.395
Yes	223 (37.8%)	123 (40.7%)	
Stage			
I + II		198 (65.6%)	
III + IV		104 (34.4%)	
Tumor T status			
≤T2		202 (66.9%)	
>T2		100 (33.1%)	
Lymph node status			
N0		293 (97.0%)	
N1 + N2		9 (3.0%)	
Metastasis			
M0		285 (94.4%)	
M1		17 (5.6%)	
Vascular invasion			
No		256 (84.8%)	
Yes		46 (15.2%)	

^†^Mann-Whitney U test or Fisher’s exact test was used between controls and patients with HCC.

**Table 2:** Distribution frequency of *SERPINB5* genotypes in 590 controls and 302 patients with HCC.

Variables	Controls (N = 590) n (%)	Patients (N = 302) n (%)	OR (95% CI)	AOR (95% CI)
rs17071138				
TT	476 (80.7%)	228 (75.5%)	1.00	1.00
TC	107 (18.1%)	70 (23.2%)	1.366 (0.972–1.919)	1.367 (0.904–2.068)
CC	7 (1.2%)	4 (1.3%)	1.193 (0.346–4.117)	0.832 (0.181–3.834)
TC + CC	114 (19.3%)	74 (24.5%)	1.355 (0.972–1.890)	1.330 (0.887–1.992)
rs3744941				
CC	244 (41.4%)	125 (41.4%)	1.00	1.00
CT	272 (46.1%)	151 (50.0%)	1.084 (0.808–1.453)	1.209 (0.849–1.721)
TT	74 (12.5%)	26 (8.6%)	0.686 (0.418–1.126)	0.687 (0.387–1.216)
CT + TT	346 (58.6%)	177 (58.6%)	0.999 (0.754–1.323)	1.081 (0.773–1.511)
rs8089104				
CC	166 (28.1%)	94 (31.1%)	1.00	1.00
CT	275 (46.6%)	150 (49.7%)	0.963 (0.698–1.329)	1.161 (0.791–1.703)
TT	149 (25.3%)	58 (19.2%)	0.687 (0.463–1.020)	0.696 (0.433–1.118)
CT + TT	424 (71.9%)	208 (68.9%)	0.866 (0.640–1.172)	0.990 (0.692–1.418)
rs2289519				
CC	234 (39.7%)	123 (40.7%)	1.00	1.00
CT	276 (46.8%)	140 (46.4%)	0.965 (0.716–1.300)	0.970 (0.680–1.384)
TT	80 (13.5%)	39 (12.9%)	0.927 (0.597–1.441)	1.056 (0.625–1.785)
CT + TT	356 (60.3%)	179 (59.3%)	0.957 (0.721–1.269)	0.989 (0.707–1.382)
rs2289520				
GG	293 (49.7%)	169 (56.0%)	1.00	1.00
GC	242 (41.0%)	122 (40.4%)	0.874 (0.655–1.166)	0.774 (0.547–1.095)
CC	55 (9.3%)	11 (3.6%)	0.347 (0.177–0.681)*	0.247 (0.113–0.543)*
GC + CC	297 (50.3%)	133 (44.0%)	0.776 (0.588–1.026)	0.666 (0.477–0.929)*
rs1455555				
AA	180 (30.5%)	85 (28.1%)	1.00	1.00
AG	295 (50.0%)	149 (49.4%)	1.070 (0.773–1.480)	1.190 (0.807–1.754)
GG	115 (19.5%)	68 (22.5%)	1.252 (0.843–1.860)	1.278 (0.795–2.055)
AG + GG	410 (69.5%)	217 (71.9%)	1.121 (0.825–1.522)	1.215 (0.842–1.753)

The odds ratios (ORs) and with their 95% confidence intervals (CIs) were estimated by logistic regression models. The adjusted odds ratios (AORs) with their 95% confidence intervals (CIs) were estimated by multiple logistic regression models after controlling for age, gender, and tobacco and alcohol consumption. *P value <  0.05 as statistically significant.

**Table 3:** Adjusted odds ratio (AOR) and 95% confidence interval (CI) of clinical status and *SERPINB5 rs2289520* genotypic frequencies in 302 HCC patients with tobacco consumption.

Variables	Genotypic frequencies			
	GG (N = 169)	GC + CC (N = 133)	OR (95% CI)	*P* value^†^
Clinical Stage				
Stage I/II	114 (67.5%)	84 (63.2%)	1.00	*P* = 0.435
Stage III/IV	55 (32.5%)	49 (36.8%)	1.209 (0.750–1.948)	
Tumor size				
≦T2	118 (69.8%)	84 (63.2%)	1.00	*P* = 0.222
>T2	51 (30.2%)	49 (36.8%)	1.350 (0.834–2.185)	
Lymph node metastasis				
No	162 (95.9%)	131 (98.5%)	1.00	*P* = 0.181
Yes	7 (4.1%)	2 (1.5%)	0.353 (0.072–1.730)	
Distant metastasis				
No	156 (92.3%)	129 (97.0%)	1.00	*P* = 0.080
Yes	13 (7.7%)	4 (3.0%)	0.372 (0.118–1.169)	
Vascular invasion				
No	141 (83.4%)	115 (86.5%)	1.00	*P* = 0.466
Yes	28 (16.6%)	18 (13.5%)	0.788 (0.415–1.497)	
Child–Pugh grade^†^				
A	122 (72.2%)	109 (82.0%)	1.00	*P* = 0.047*
B or C	47 (27.8%)	24 (18.0%)	0.527 (0.328–0.996)	
HBsAg				
Negative	107 (63.3%)	70 (52.6%)	1.00	*P* = 0.061
Positive	62 (36.7%)	63 (47.4%)	1.553 (0.978–2.466)	
Anti–HCV				
Negative	79 (46.7%)	77 (57.9%)	1.00	*P* = 0.054
Positive	90 (53.3%)	56 (42.1%)	0.638 (0.404–1.009)	
Liver cirrhosis				
Negative	30 (17.8%)	35 (26.3%)	1.00	*P* = 0.072
Positive	139 (82.2%)	98 (73.7%)	0.604 (0.348–1.049)	

The ORs with analyzed by their 95% CIs were estimated by logistic regression models. >T2 indicated the multiple tumor more than 5 cm or tumor involving a major branch of the portal or hepatic vein(s). *P value < 0.05 as statistically significant.

^†^Child-Pugh grades indicate the severity of cirrhosis: A = 5–6 points, B = 7–9 points and C = 10–15 points.

**Table 4:** The estimated haplotype frequencies of six examined polymorphisms in *SERPINB5* gene and the corresponding risk for HCC.

Block 1			Controls (N = 1180) n (%)	Patients (N = 604) n (%)	OR (95% CI)	*P*value
rs17071138 T/C	rs3744941 C/T	rs8089104 C/T				
T	C	C	505 (42.8%)	256 (42.4%)	Reference	
T	T	T	417 (35.4%)	196 (32.5%)	0.927 (0.739–1.163)	0.514
T	C	T	137 (11.6%)	68 (11.2%)	0.979 (0.706–1.358)	0.899
C	C	C	102 (8.6%)	75 (12.4%)	1.450 (1.039–2.025)	0.029
Others#			19 (1.6%)	9 (1.5%)	0.934 (0.417–2.095)	0.869
Block 2			Controls (N = 1180) n (%)	Patients (N = 604) n (%)	OR (95% CI)	*P*value
rs2289519 T/C	rs2289520 G/C	rs1455555 A/G				
T	G	A	343 (29.1%)	186 (30.8%)	Reference	
T	C	A	332 (28.1%)	134 (22.2%)	0.744 (0.569–0.974)	0.031
C	G	A	264 (22.4%)	127 (21.0%)	0.887 (0.673–1.170)	0.396
T	G	G	167 (14.2%)	89 (14.7%)	0.983 (0.719–1.344)	0.913
C	C	G	54 (4.6%)	58 (9.6%)	1.981 (1.313–2.989)	0.001
Others#			20 (1.6%)	10 (1.7%)	0.922 (0.423–2.011)	0.838

^#^Block 1 Others: CCT (18; control: 16; patient: 2), CTT (3; control: 3; patient: 0), TTC (6; control: 0; patient: 6), CTC (1; control: 0; patient: 1).

^#^Block 2 Others: CCA (23; control: 15; patient: 8), CAG (5; control: 5; patient: 0), TCG (2; control: 0; patient: 2).

## References

[b1] Torre L. A. *et al.* Global cancer statistics, 2012. CA Cancer J Clin 65, 87–108 (2015). 25651787 10.3322/caac.21262

[b2] Bosch F. X., Ribes J., Díaz M. & Cléries R. Primary liver cancer: Worldwide incidence and trends. Gastroenterology 127, S5–S16 (2004). 15508102 10.1053/j.gastro.2004.09.011

[b3] Tsai W. L. & Chung R. T. Viral hepatocarcinogenesis. Oncogene 29, 2309–2324 (2010). 20228847 10.1038/onc.2010.36 PMC3148694

[b4] Farazi P. A. & DePinho R. A. Hepatocellular carcinoma pathogenesis: from genes to environment. Nat Rev Cancer 6, 674–687 (2006). 16929323 10.1038/nrc1934

[b5] Wang L. Y. *et al.* Risk of hepatocellular carcinoma and habits of alcohol drinking, betel quid chewing and cigarette smoking: a cohort of 2416 HBsAg-seropositive and 9421 HBsAg-seronegative male residents in Taiwan. Cancer Causes Control 14, 241–250 (2003). 12814203 10.1023/a:1023636619477

[b6] Altamirano J. & Bataller R. Cigarette smoking and chronic liver diseases. Gut 59, 1159–1162 (2010). 20650922 10.1136/gut.2008.162453

[b7] Wong V. W. *et al.* Clinical scoring system to predict hepatocellular carcinoma in chronic hepatitis B carriers. J Clin Oncol 28, 1660–1665 (2010). 20194845 10.1200/JCO.2009.26.2675

[b8] Asahina Y. *et al.* Effect of aging on risk for hepatocellular carcinoma in chronic hepatitis C virus infection. Hepatology 52, 518–527 (2010). 20683951 10.1002/hep.23691

[b9] Lee C. M. *et al.* Age, gender, and local geographic variations of viral etiology of hepatocellular carcinoma in a hyperendemic area for hepatitis B virus infection. Cancer 86, 1143–1150 (1999). 10506697 10.1002/(sici)1097-0142(19991001)86:7<1143::aid-cncr7>3.0.co;2-z

[b10] Chan S. L., Wong V. W., Qin S. & Chan H. L. Infection and Cancer: The Case of Hepatitis B. J Clin Oncol 34, 83–90 (2016). 26578611 10.1200/JCO.2015.61.5724

[b11] Zou Z. *et al.* Maspin, a serpin with tumor-suppressing activity in human mammary epithelial cells. Science 263, 526–529 (1994). 8290962 10.1126/science.8290962

[b12] Blacque O. E. & Worrall D. M. Evidence for a direct interaction between the tumor suppressor serpin, maspin, and types I and III collagen. J Biol Chem 277, 10783–10788 (2002). 11788595 10.1074/jbc.M110992200

[b13] Sheng S. *et al.* Maspin acts at the cell membrane to inhibit invasion and motility of mammary and prostatic cancer cells. Proc Natl Acad Sci USA 93, 11669–11674 (1996). 8876194 10.1073/pnas.93.21.11669 PMC38116

[b14] Vecchi M. *et al.* Breast cancer metastases are molecularly distinct from their primary tumors. Oncogene 27, 2148–2158 (2008). 17952122 10.1038/sj.onc.1210858

[b15] Teoh S. S., Whisstock J. C. & Bird P. I. Maspin (SERPINB5) is an obligate intracellular serpin. J Biol Chem 285, 10862–10869 (2010). 20123984 10.1074/jbc.M109.073171 PMC2856292

[b16] Bal N., Kocer N. E., Ertorer M. E., Canpolat E. T. & Kayaselcuk F. Maspin, E-selectin, and P-selectin expressions in papillary thyroid carcinomas and their correlation with prognostic parameters. Pathology, research and practice 204, 743–750 (2008). 10.1016/j.prp.2008.04.016 18597952

[b17] Berardi R. *et al.* Role of maspin in cancer. Clin Transl Med 2, 8 (2013). 23497644 10.1186/2001-1326-2-8 PMC3602294

[b18] Bascones-Martinez A. *et al.* Differences in the expression of five senescence markers in oral cancer, oral leukoplakia and control samples in humans. Oncol Lett 3, 1319–1325 (2012). 22783442 10.3892/ol.2012.649 PMC3392562

[b19] Nickoloff B. J. *et al.* Tumor suppressor maspin is up-regulated during keratinocyte senescence, exerting a paracrine antiangiogenic activity. Cancer Res 64, 2956–2961 (2004). 15126325 10.1158/0008-5472.can-03-2388

[b20] Sheng S. A role of novel serpin maspin in tumor progression: the divergence revealed through efforts to converge. J Cell Physiol 209, 631–635 (2006). 17001674 10.1002/jcp.20786

[b21] Law R. H. *et al.* The high resolution crystal structure of the human tumor suppressor maspin reveals a novel conformational switch in the G-helix. J Biol Chem 280, 22356–22364 (2005). 15760906 10.1074/jbc.M412043200

[b22] Feitelson M. A. Parallel epigenetic and genetic changes in the pathogenesis of hepatitis virus-associated hepatocellular carcinoma. Cancer Lett 239, 10–20 (2006). 16154256 10.1016/j.canlet.2005.07.009

[b23] Thorgeirsson S. S. & Grisham J. W. Molecular pathogenesis of human hepatocellular carcinoma. Nat Genet 31, 339–346 (2002). 12149612 10.1038/ng0802-339

[b24] Li M. X. *et al.* Prognostic Role of Glasgow Prognostic Score in Patients With Hepatocellular Carcinoma: A Systematic Review and Meta-Analysis. Medicine (Baltimore) 94, e2133 (2015). 26656342 10.1097/MD.0000000000002133 PMC5008487

[b25] Genomes Project C. *et al.* An integrated map of genetic variation from 1,092 human genomes. Nature 491, 56–65 (2012). 23128226 10.1038/nature11632 PMC3498066

[b26] Barrett J. C., Fry B., Maller J. & Daly M. J. Haploview: analysis and visualization of LD and haplotype maps. Bioinformatics 21, 263–265 (2005). 15297300 10.1093/bioinformatics/bth457

[b27] Zou Z. *et al.* p53 regulates the expression of the tumor suppressor gene maspin. J Biol Chem 275, 6051–6054 (2000). 10692390 10.1074/jbc.275.9.6051

[b28] Kim S., Han J., Kim J. & Park C. Maspin expression is transactivated by p63 and is critical for the modulation of lung cancer progression. Cancer Res 64, 6900–6905 (2004). 15466179 10.1158/0008-5472.CAN-04-1657

[b29] Kellis M. *et al.* Defining functional DNA elements in the human genome. Proc Natl Acad Sci USA 111, 6131–6138 (2014). 24753594 10.1073/pnas.1318948111 PMC4035993

[b30] Dreos R., Ambrosini G., Cavin Perier R. & Bucher P. EPD and EPDnew, high-quality promoter resources in the next-generation sequencing era. Nucleic Acids Res 41, D157–164 (2013). 23193273 10.1093/nar/gks1233 PMC3531148

[b31] Al-Ayyoubi M., Gettins P. G. & Volz K. Crystal structure of human maspin, a serpin with antitumor properties: reactive center loop of maspin is exposed but constrained. J Biol Chem 279, 55540–55544 (2004). 15501821 10.1074/jbc.M409957200

[b32] Yin S., Ding F. & Dokholyan N. V. Eris: an automated estimator of protein stability. Nat Methods 4, 466–467 (2007). 17538626 10.1038/nmeth0607-466

[b33] Baert-Desurmont S. *et al.* Clinical relevance of 8q23, 15q13 and 18q21 SNP genotyping to evaluate colorectal cancer risk. European journal of human genetics : EJHG 24, 99–105 (2016). 25873010 10.1038/ejhg.2015.72 PMC4795220

[b34] Cai T. *et al.* Loss of heterozygosis on chromosome 18q21-23 and muscle-invasive bladder cancer natural history. Oncol Lett 10, 2569–2573 (2015). 26622891 10.3892/ol.2015.3616 PMC4579988

[b35] Shiiba M. *et al.* Down-regulated expression of SERPIN genes located on chromosome 18q21 in oral squamous cell carcinomas. Oncology reports 24, 241–249 (2010). 20514468 10.3892/or_00000852

[b36] Kim M., Ju H., Lim B. & Kang C. Maspin genetically and functionally associates with gastric cancer by regulating cell cycle progression. Carcinogenesis 33, 2344–2350 (2012). 22962304 10.1093/carcin/bgs280

[b37] Nakagawa M. *et al.* Maspin expression and its clinical significance in non-small cell lung cancer. Ann Surg Oncol 13, 1517–1523 (2006). 17009165 10.1245/s10434-006-9030-z

[b38] Abd El-Maqsoud N. M. & Tawfiek E. R. Loss of Maspin Expression in Bladder Cancer: Its Relationship with p53 and Clinicopathological Parameters. J Egypt Natl Canc Inst 22, 1–12 (2010). 21503001

[b39] Kim J. H. *et al.* Nuclear maspin expression correlates with the CpG island methylator phenotype and tumor aggressiveness in colorectal cancer. Int J Clin Exp Pathol 8, 1920–1928 (2015). 25973084 PMC4396253

[b40] Meng H. *et al.* Association between SNPs in Serpin gene family and risk of esophageal squamous cell carcinoma. Tumour biology : the journal of the International Society for Oncodevelopmental Biology and Medicine 36, 6231–6238 (2015). 25775950 10.1007/s13277-015-3308-3

[b41] Jang H. L. *et al.* Maspin polymorphism associated with apoptosis susceptibility and *in vivo* tumorigenesis. Int J Mol Med 22, 333–338 (2008). 18698492

[b42] Sastre J. *et al.* Clinical guideline SEOM: hepatocellular carcinoma. Clin Transl Oncol 17, 988–995 (2015). 26607931 10.1007/s12094-015-1451-3 PMC4689753

[b43] Bruix J. & Sherman M. & American Association for the Study of Liver, D. Management of hepatocellular carcinoma: an update. Hepatology 53, 1020–1022 (2011). 21374666 10.1002/hep.24199 PMC3084991

[b44] Zavras A. I., Yoon A. J., Chen M. K., Lin C. W. & Yang S. F. Metallothionein-1 genotypes in the risk of oral squamous cell carcinoma. Ann Surg Oncol 18, 1478–1483 (2011). 21128001 10.1245/s10434-010-1431-3

[b45] Glad C. A., Johannsson G., Carlsson L. M. & Svensson P. A. Rapid and high throughput genotyping of the growth hormone receptor exon 3 deleted/full-length polymorphism using a tagSNP. Growth Horm IGF Res 20, 270–273 (2010). 20219401 10.1016/j.ghir.2010.02.004

[b46] Ward L. D. & Kellis M. HaploReg v4: systematic mining of putative causal variants, cell types, regulators and target genes for human complex traits and disease. Nucleic Acids Res 44, D877–881 (2016). 26657631 10.1093/nar/gkv1340 PMC4702929

[b47] Consortium G. T. The Genotype-Tissue Expression (GTEx) project. Nat Genet 45, 580–585 (2013). 23715323 10.1038/ng.2653 PMC4010069

[b48] Pazin M. J. Using the ENCODE Resource for Functional Annotation of Genetic Variants. Cold Spring Harb Protoc 2015, 522–536 (2015). 25762420 10.1101/pdb.top084988 PMC4521767

[b49] Cerami E. *et al.* The cBio cancer genomics portal: an open platform for exploring multidimensional cancer genomics data. Cancer Discov 2, 401–404 (2012). 22588877 10.1158/2159-8290.CD-12-0095 PMC3956037

[b50] Goldman M. *et al.* The UCSC Cancer Genomics Browser: update 2015. Nucleic Acids Res 43, D812–817 (2015). 25392408 10.1093/nar/gku1073 PMC4383911

[b51] Rose P. W. *et al.* The RCSB Protein Data Bank: views of structural biology for basic and applied research and education. Nucleic Acids Res 43, D345–356 (2015). 25428375 10.1093/nar/gku1214 PMC4383988

