# Supplementary material for: Serpin peptidase inhibitor (SERPINB5) haplotypes are associated with susceptibility to hepatocellular carcinoma
Source: Sci Rep. 2016 May 25;6:26605. doi: 10.1038/srep26605 (PMC4879545; doi:10.1038/srep26605)
Supplement: Supplementary Information [file srep26605-s1.pdf]

Title

Serpin peptidase inhibitor (*SERPINB5*) haplotypes are associated with susceptibility to hepatocellular carcinoma

Author list and affiliations

Shun-Fa Yang[1,2], Chao-Bin Yeh[3,4], Ying-Erh Chou[2,5], Hsiang-Lin Lee[1,6], and Yu-Fan Liu[7,8*]

[1]Institute of Medicine, Chung Shan Medical University, Taichung, Taiwan

[2]Department of Medical Research, Chung Shan Medical University Hospital, Taichung, Taiwan

[3]Department of Emergency Medicine, School of Medicine, Chung Shan Medical University, Taichung, Taiwan

[4]Department of Emergency Medicine, Chung Shan Medical University Hospital, Taichung, Taiwan

[5]School of Medicine, Chung Shan Medical University, Taichung, Taiwan

[6]Deptartment of Surgery, Chung Shan Medical University Hospital, Taichung, Taiwan

[7]Department of Biomedical Sciences, College of Medicine Sciences and Technology, Chung Shan Medical University, Taichung, Taiwan

[8]Division of Allergy, Department of Pediatrics, Chung-Shan Medical University Hospital, Taichung, Taiwan

***Address correspondence to:** Yu-Fan Liu, Ph.D., Department of Biomedical Sciences, College of Medicine Sciences and Technology, Chung Shan Medical University, 110 Sec. 1, Chien-Kuo N. Road, Taichung 40201, Taiwan. Telephone: +886-4-24730022 ext. 11819; Fax: +886-4-24751101

E-mail: yfliu@csmu.edu.tw

Table S1 | Exon and intron structure of *SERPINB5* gene in Homo sapiens and the feature of SNPs of *SERPINB5*, which were used to analyze in this study

	Exon (chromosome position)					
	**Promoter** (61,143,908)#	**Promoter** (61,144,053)	**Intron** (61,148,149)	**6** (61,160,287)	**6** (61,160,320)	**8** (61,170,782)
**nucleotide change**	T>C	C>T	C>T	C>T	G>C	A>G
**mRNA position†**	-	-		668	701	1,097
**Protein position‡**				176	187	319
**dbSNP (rs number)**	rs17071138	rs3744941	rs8089104	rs2289519	rs2289520	rs1455555
**Function**	-	-	-	nonsynonymous	nonsynonymous	nonsynonymous
**dbSNP allele**	-	-	-	TCT>CCT	GTC>CTC	ATC>GTC
**Protein residue**	-	-	-	Ser>Pro	Val>Leu	Ile>Val
**Codon position**	-	-	-	1	1	1
**Heterozygous (%)**	22.2	46.5	51.1	51.2	32.6	62.8

GRCh37.p13
† NM_002693.4
‡ NP_002630.2

TABLE S2 | Association of *SERPINB5* genotypic frequencies with HCC laboratory status

Characteristic	α-Fetoprotein† (ng/mL)	AST† (IU/L)	ALT† (IU/L)	AST/ALT† ratio
**rs1455555**				
AA	4111.4 ± 1892.4	132.5 ± 21.4	108.1 ± 18.7	1.68 ± 0.25
AG+GG	2557.8 ± 833.0	152.9 ± 23.1	128.8 ± 17.8	1.45 ± 0.08
P value	0.384	0.604	0.503	0.242
**rs17071138**				
TT	2661.6 ± 854.9	128.5 ± 16.3	113.4 ± 13.7	1.46 ± 0.10
TC+CC	4022.7 ± 1939.1	204.4 ± 51.3	152.5 ± 37.4	1.67 ± 0.18
P value	0.465	0.064	0.224	0.305
**rs2289519**				
CC	4701.5 ± 1746.8	180.3 ± 37.1	133.8 ± 24.5	1.47 ± 0.08
CT+TT	1822.5 ± 609.7	124.3 ± 15.2	115.5 ± 16.2	1.54 ± 0.14
P value	0.077	0.119	0.517	0.682
**rs2289520**				
GG	2340.7 ± 795.2	139.8 ± 20.8	129.3 ± 17.9	1.39 ± 0.09
GC+CC	3826.6 ± 1511.8	156.4 ± 30.2	115.0 ± 21.7	1.67 ± 0.17
P value	0.358	0.640	0.609	0.118
**rs3744941**				
CC	3630.3 ± 1559.0	157.2 ± 30.8	126.2 ± 23.1	1.47 ± 0.08
CT+TT	2546.5 ± 810.8	140.0 ± 20.8	120.7 ± 17.1	1.54 ± 0.14
P value	0.506	0.632	0.847	0.667
**rs8089104**				
CC	2289.6 ± 1192.9	183.8 ± 41.3	138.1 ± 29.8	1.50 ± 0.10
CT+TT	3313.9 ± 1030.6	130.5 ± 17.5	116.1 ± 14.9	1.52 ± 0.12
P value	0.554	0.763	0.463	0.950

Mann-Whitney U test was used between two groups.
† Mean ± S.E.

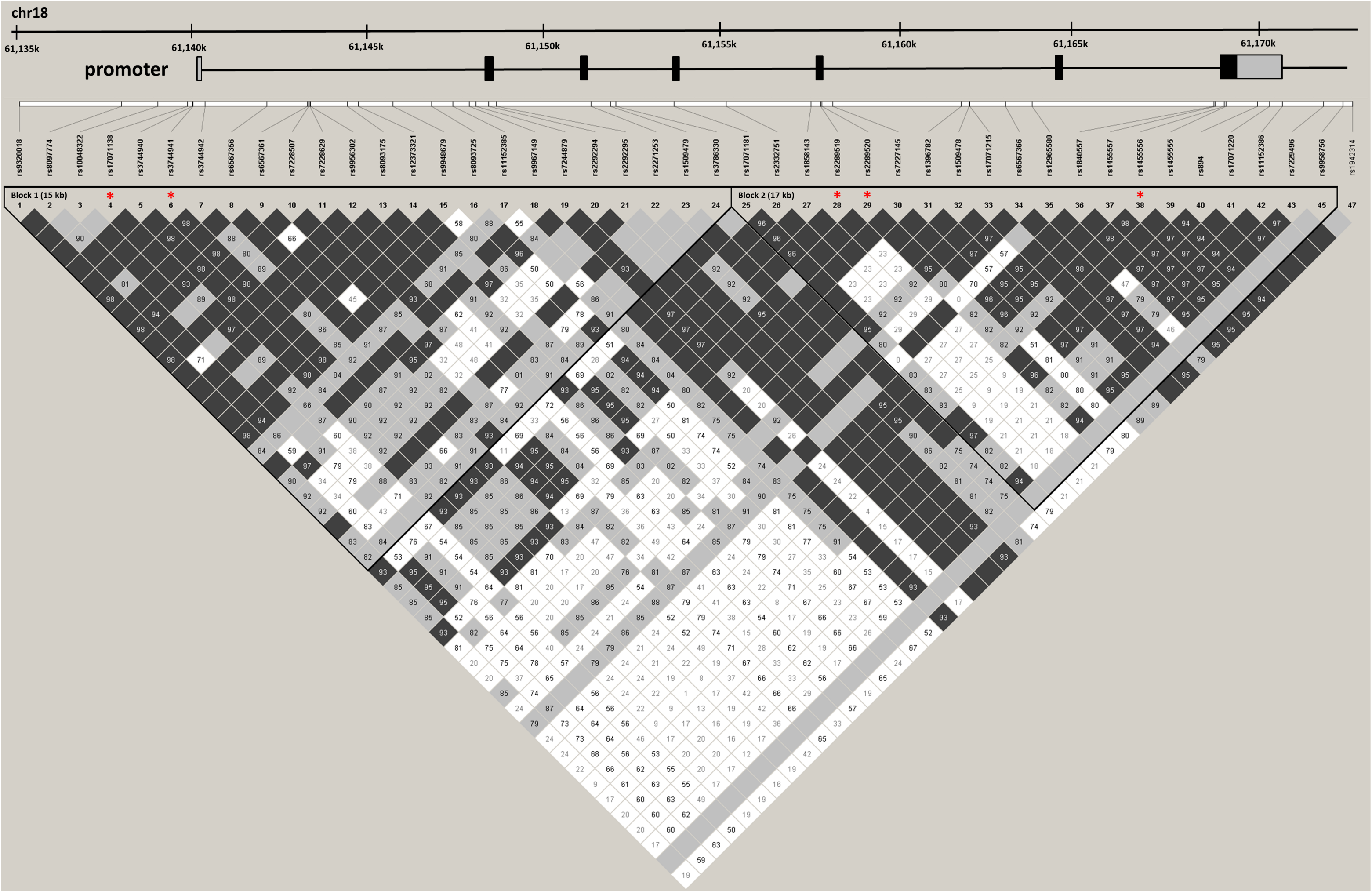

Figure S1 | Linkage disequilibrium map constructed from 45 selected SNP tags in the SERPINB5 gene. In the LD map, SNP-pairwise correlation coefficients and Hedrick's multiallelic D') in the East Asian population (HCB+JPT) are shown in black squares when D'=1.0 and white squares when D'=0. The "4 Gamete" conventional grey scale is used to display the LDs shown in black through grey (the colour intensity decreases with the decrease in the D' value) generated by Haploview version 4.2. SNPs located within and around the SERPINB5 gene were plotted against the chromosomal positions chr.18: 61,135,000 to 61,180,000 (HapMap version 3 release 27). Exons, introns and untranslated regions are indicated with filled boxes, unfilled boxes and thin lines from the 5'- to 3'- end of this gene, respectively. The red stars indicate the selected genotyping of the SERPINB5 SNP polymorphisms.

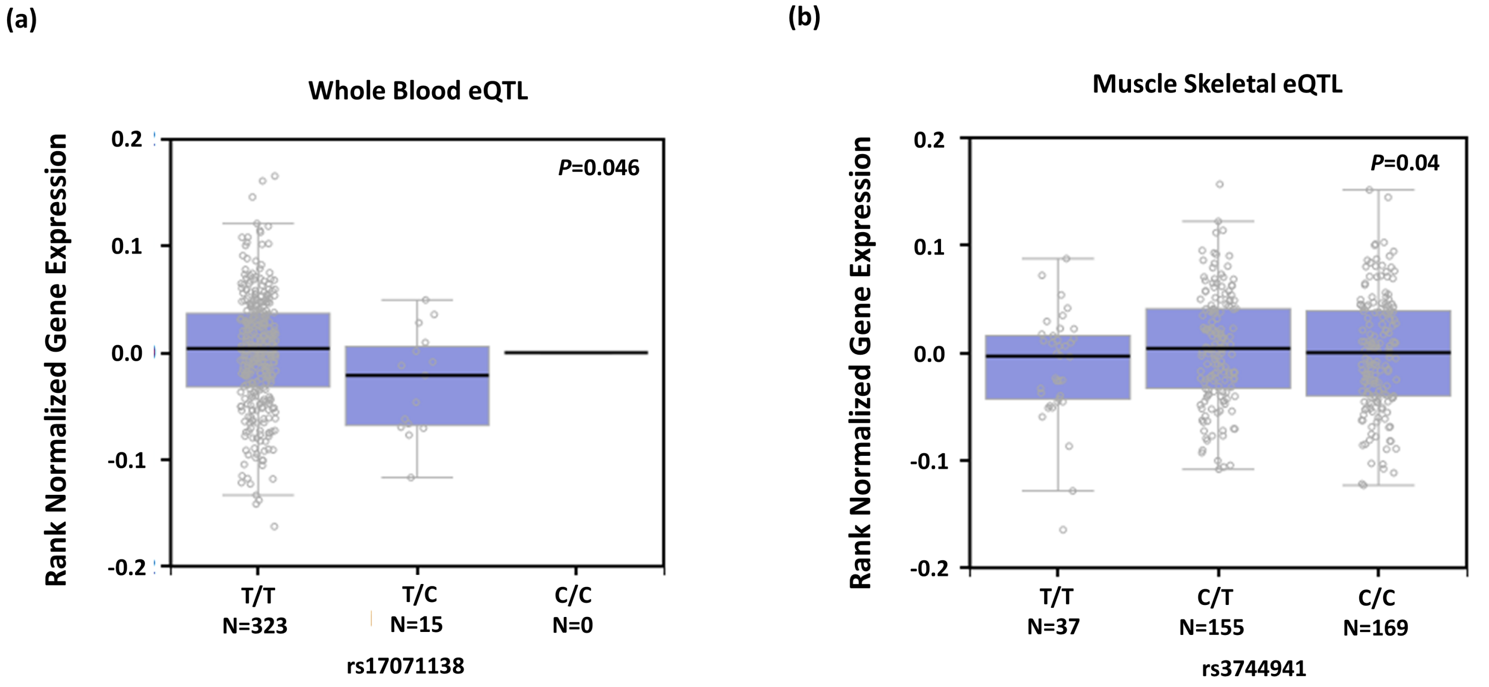

Figure S2 | Expression of quantitative trait loci of the HCC risk-associated haplotype and the features of the *SERPINB5* gene SNPs (NM_002639) analysed in this study. Expression quantitative trait loci associations between **(a)** rs17071138 and **(b)** rs3744941 with *SERPINB5* expression in the whole blood and muscle_skeletal (GTEx data set), respectively. Numbers in parentheses indicate the numbers of cases.
